# Enhancing daily streamflow simulation using the coupled SWAT-BiLSTM approach for climate change impact assessment in Hai-River Basin

**DOI:** 10.1038/s41598-023-42512-4

**Published:** 2023-09-13

**Authors:** Xianqi Zhang, Yu Qi, Fang Liu, Haiyang Li, Shifeng Sun

**Affiliations:** 1 Water Conservancy College, North China University of Water Resources and Electric Power, Zhengzhou, 450046 China; 2 Collaborative Innovation Center of Water Resources Efficient Utilization and Protection Engineering, Zhengzhou, 450046 China; 3 Technology Research Center of Water Conservancy and Marine Traffic Engineering, Henan Province, Zhengzhou, 450046 China

**Keywords:** Climate change, Hydrology

## Abstract

Against the backdrop of accelerated global climate change and urbanization, the frequency and severity of flood disasters have been increasing. In recent years, influenced by climate change, the Hai-River Basin (HRB) has experienced multiple large-scale flood disasters. During the widespread extraordinary flood event from July 28th to August 1st, 2023, eight rivers witnessed their largest floods on record. These events caused significant damage and impact on economic and social development. The development of hydrological models with better performance can help researchers understand the impacts of climate change, provide risk information on different disaster events within watersheds, support decision-makers in formulating adaptive measures, urban planning, and improve flood defense mechanisms to address the ever-changing climate environment. This study examines the potential for enhancing streamflow simulation accuracy in the HRB located in Northeast China by combining the physically-based hydrological model with the data-driven model. Three hybrid models, SWAT-D-BiLSTM, SWAT-C-BiLSTM and SWAT-C-BiLSTM with SinoLC-1, were constructed in this study, in which SWAT was used as a transfer function to simulate the base flow and quick flow generation process based on weather data and spatial features, and BiLSTM was used to directly predict the streamflow according to the base flow and quick flow. In the SWAT-C-BiLSTM model, SWAT parameters with *P* values less than 0.4 in each hydrological station-controlled watershed were calibrated, while the SWAT-D-BiLSTM model did not undergo calibration. Additionally, this study utilizes both 30 m resolution land use and land cover (LULC) map and the first 1 m resolution LULC map SinoLC-1 as input data for the models to explore the impact on streamflow simulation performance. Among five models, the NSE of SWAT-C-BiLSTM with SinoLC-1 reached 0.93 and the R^2^ reached 0.95 during the calibration period, and both of them stayed at 0.92 even in the validation period, while the NSE and R^2^ of the other four models were all below 0.90 in the validation period. The potential impact of climate change on streamflow in the HRB was evaluated by using predicted data from five global climate models from CMIP6 as input for the best-performing SWAT-C-BiLSTM with SinoLC-1. The results indicate that climate change exacerbates the uneven distribution of streamflow in the HRB, particularly during the concentrated heavy rainfall months of July and August. It is projected that the monthly streamflow in these two months will increase by 34% and 49% respectively in the middle of this century. Furthermore, it is expected that the annual streamflow will increase by 5.6% to 9.1% during the mid-century and by 6.7% to 9.3% by the end of the century. Both average streamflow and peak streamflow are likely to significantly increase, raising concerns about more frequent urban flooding in the capital economic region within the HRB.

## Introduction

Climate change is a global issue of widespread concern in today's society. Over the past century, the global climate has undergone changes primarily characterized by warming, resulting in increased evaporation, atmospheric water vapor content, and changes in precipitation patterns and intensity. These changes have exacerbated the global hydrological cycle and have had a significant impact on streamflow^1,2^. As an integral part of the hydrological cycle, changes in streamflow directly affect human life and production activities^3^. Accurate estimation of streamflow changes is of great significance for water resource management planning and flood risk assessment^4^. Over the past few years, the global phenomenon of climate change has resulted in heightened occurrences and intensification of floods across numerous regions worldwide^5,6^. Climate change, coupled with rapid urbanization, has exacerbated flooding disasters in coastal and inland areas. The drainage systems of many cities are inadequate to cope with these increasingly severe floods, and the risks associated with more frequent extreme rainfall events are becoming imminent. The Hai-River Basin (HRB) is one of the seven major river basins in China, covering 3.3% of the country's land area. It exhibits complex spatial characteristics and is frequently affected by subtropical high-pressure systems and typhoons, resulting in frequent occurrence of floods during the rainy season. For example, in July 2021, the basin experienced an extreme rainfall event with a cumulative precipitation of 250–350 mm, reaching 500–657 mm in some areas of Zhengzhou City. The maximum hourly rainfall intensity reached 201.9 mm/h, setting a record for the highest hourly precipitation intensity in large and medium-sized cities globally^7^. The HRB had already experienced a rainfall event from July 10th to 12th, saturating the soil, and on the 17th, rainfall resumed, causing widespread runoff throughout the basin. The rapid inflow of rainwater into the rivers led to a sharp rise in river levels, with peak flow exceeding 1890 m^3^/s. This event resulted in an economic loss of 120.06 billion CNY and 398 deaths (Ministry of Emergency Management, PRC, 2021). To help researchers understand the impacts of climate change, provide risk information on different disaster events within watersheds, support decision-makers in formulating adaptive measures and urban planning, and improve flood defense mechanisms to address the ever-changing climate environment, there is a need for hydrological models with better performance to simulate streamflow ^8^.

Physically-based hydrological models utilize physical principles and equations to describe hydrological processes. These models are based on fundamental physics laws such as fluid mechanics, thermodynamics, and soil water movement to quantitatively simulate the hydrological cycle and water movement within a watershed. Hydrological prediction models built using multidimensional data such as regional hydrological and environmental variables, climate, topography, and vegetation have been increasingly applied in practical water environment management and disaster reduction efforts. In recent decades, numerous physically-based hydrological models have emerged and gained global recognition for their widespread application, such as Xinanjiang^9^, TOPMODEL^10^, VIC-2L^11^, SWAT^12^, and SWMM^13^. In spite of the diverse physically-based models and their inherent process descriptions, their application encounters uncertainties across three key aspects: model structure, accuracy of input data, and parameter calibration^14–16^. One notable challenge in the calibration process of these models is equifinality, where different parameter combinations can yield similar hydrological simulation results^17^. Furthermore, conceptual hydrological models exhibit limitations in accurately predicting peak flow, particularly during significant rainfall events. For instance, Pereira et al.^18^ found that SWAT underestimated the peak flow in the Pomba River Basin, and Yuan et al.^19^ found that SWMM underestimated the peak flow in two major rainfall events in Elizabeth City. Furthermore, many previous studies have indicated that physically-based hydrological models often underestimate peak flow in simulations^20–22^.

Data-driven models are modeling methods based on data analysis and pattern recognition. They learn and analyze a large amount of input data to discover patterns, trends, and correlations within the data, thus establishing the relationship between input and output data. The construction of such models usually does not require a detailed understanding of the underlying system but relies on the inherent characteristics of the data for prediction and decision-making. Data-driven models can be linear or nonlinear. Linear data-driven models aim to establish a linear relationship between input and output data, such as linear regression models. Nonlinear data-driven models, on the other hand, are more flexible and capable of capturing complex nonlinear relationships between inputs and outputs, such as neural networks and support vector machines. Currently, data-driven models have found extensive application in hydrological research^23,24^. As a type of data-driven model, Long Short-Term Memory (LSTM) has been applied in various hydrological applications, including precipitation forecasting^25^, groundwater level prediction^26^, and streamflow prediction^27^. In recent times, the effectiveness of Bidirectional LSTM (BiLSTM), which incorporates two LSTMs operating in opposite directions, has been demonstrated to surpass that of conventional unidirectional LSTM approaches. For instance, Zhang et al.^28^ discovered that BiLSTM exhibited superior performance compared to unidirectional LSTM when it came to forecasting precipitation variations. Nevertheless, the rainfall-runoff process represents a intricate nonlinear phenomenon that is subject to the influence of diverse spatial variable factors, including topographic disparities, land use and land cover (LULC) classifications, soil types, and prevailing weather conditions. For instance, Jiang et al.^29^ noted that as the center of a heavy rainstorm approaches the outlet, the water level at the watershed outlet rapidly rises to its maximum level, albeit for a brief period. Existing data-driven hydrological models fail to consider the profound influence of spatial variations on changes in streamflow. They overly rely on time series of different meteorological variables as training data, especially when dealing with large-scale watersheds characterized by complex spatial features^30^.

In recent years, numerous research studies have focused on the integration of physically-based conceptual hydrological models with data-driven models. The objective of this integration is to leverage the unique advantages of each approach and improve the accuracy of streamflow simulations. These coupled approaches involve utilizing the streamflow simulation results obtained from the conceptual models as input for the data-driven models. This integration enhances the data-driven models' capability to accurately simulate peak flows^31–33^. Chen et al.^34^ coupled the Soil & Water Assessment Tool (SWAT) model with the Long Short-Term Memory (LSTM) model to simulate daily streamflow in the Jiulong River Watershed (JRW) located in the southeastern part of Fujian Province, China. They found that the SWAT-LSTM coupled model not only provided more satisfactory performance in data-rich watersheds compared to the standalone SWAT model but also accurately reproduced streamflow sequences for any period in data-scarce similar watersheds. This demonstrated the potential of coupling conceptual hydrological models with machine learning techniques for streamflow simulation in data-scarce watersheds. However, the LSTM model is trained only through forward propagation, and this approach fails to adequately capture the potential linear or nonlinear relationships between the explanatory and target variables, resulting in lower utilization of training data^35^. BiLSTM addresses this by stacking two reversed LSTMs to simultaneously analyze the past and future correlation information between explanatory and target variables at each time step. This improved understanding of the current sequence position helps reduce errors in identifying underlying relationships. In certain datasets, the correlation information between explanatory and target variables might span a long temporal distance, leading to the gradient vanishing and exploding problems in LSTM. BiLSTM, however, is better equipped to capture such long-range dependencies^36^. Therefore, we introduce the novel SWAT-C-BiLSTM, aiming to explore its feasibility in enhancing streamflow simulation performance for the first time.

Physically-based conceptual hydrological models encounter uncertainty due to the equifinality issue during calibration, while data-driven models are unable to identify the effects of spatial variability characteristics. This study aims to couple a high-performance BiLSTM model with the SWAT model to overcome inherent limitations of both models and enhance the simulation performance of daily streamflow. Coupled Model Intercomparison Project Phase 6 (CMIP6) is an international project conducted by the climate science community, aimed at providing a unified reference dataset of global climate models. In this study, the predicted changes in rainfall and temperature under different emission scenarios from five GCMs in the CMIP6 were used as input data for the model to assess the potential impacts of climate change on hydrological processes in the HRB.

## Materials and methods

### Study area

**Figure 1:**
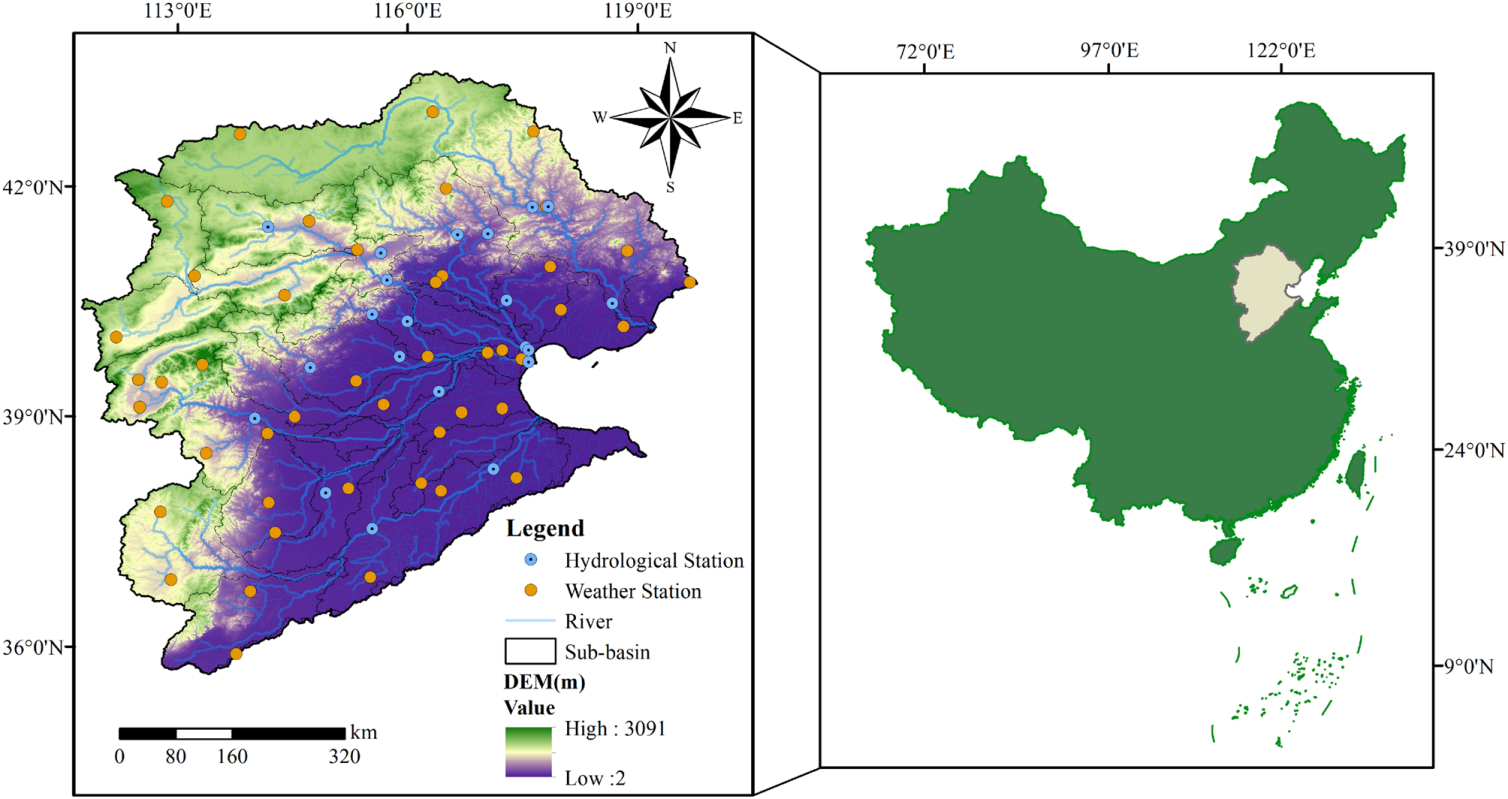
Overview of the study area. The figure is created using ArcMap 10.2, www.arcgis.com.

The HRB (121°-120°E, 35°-43°N) is located in the northeastern part of the People's Republic of China. The Hai River is formed by the convergence of five major tributaries: Zhangwei River, Ziya River, Daqing River, Yongding River, and Beiyun River. It flows for 1031 km from northwest to southeast before entering the Bohai Sea in China. The total basin area of the Hai River is 320,000 square kilometers, with 189,600 square kilometers (approximately 59%) covered by mountains and plateaus, and 131,000 square kilometers (approximately 41%) covered by plains. The HRB spans eight provinces, including Beijing, Tianjin, Hebei, Shanxi, Henan, Shandong, Inner Mongolia, and Liaoning, with a total coastline length of 920 km. The terrain in the entire basin varies greatly, with elevations ranging from 5 m in the southeastern plains to over 3000 m in the western/southwestern/northwestern mountainous areas (Fig. 1).

The HRB belongs to the temperate East Asian monsoon climate region and exhibits significant continental climate characteristics. The average annual precipitation in the basin ranges from 400 to 700 mm, with uneven distribution of rainfall. The southern foothills of the Yanshan Mountains and the eastern foothills of the Taihang Mountains receive the highest precipitation, forming an arc-shaped rainy belt. The average daily maximum and minimum temperatures hover around 23 °C and 1.5 °C, respectively, with heavy rainfall typically concentrated in July and August^37^. On one hand, most of the rivers in the basin have short lengths and steep gradients, making them prone to sudden heavy rainfall and flash floods. Some river sections face obstacles to flood discharge, leading to frequent flood disasters. On the other hand, the HRB suffers from water scarcity, excessive groundwater extraction, frequent droughts, and it serves as a major water supply area for the South-to-North Water Diversion Project. Soil erosion and water shortage issues are prominent in the region.

### Data description

When modeling water environment using the SWAT model, various data including topography, LULC dataset, soil properties, weather, and streamflow are required. In this study, we acquired relevant data for the HRB from different sources for modeling purposes (Table 1). To investigate the impact of LULC data at different resolutions on streamflow prediction, this study utilized 30 m resolution LULC data from the Resource and Environment Science and Data Center (RESDC) and very-high-resolution (VHR) LULC data SinoLC-1 from the State Key Laboratory of Information Engineering in Surveying, Mapping and Remote Sensing (LIESMARS), Wuhan University^38^.

**Table 1:** Data inputs.

Data	Data source
DEM	https://www.gscloud.cn/
LULC maps (30 m)	https://www.resdc.cn/ (CNLUCC)
LULC maps (1 m)	https://doi.org/10.5281/zenodo.7821068/ (SinoLC-1)
Soil maps	Institute of Soil Science, Chinese Academy of Sciences
River networks	National Geomatics Center of China (NGCC)
Daily rainfall records (Jan 2015–Dec 2022) (46 stations)	National Oceanic and Atmospheric Administration (NOAA) https://www.noaa.gov/
Daily temperature records (Jan 2015–Dec 2022) (46 stations)	
Daily streamflow records (Jan 2015–Dec 2022) (21 stations)	Ministry of Water Resources of the People's Republic of China

**Figure 2:**
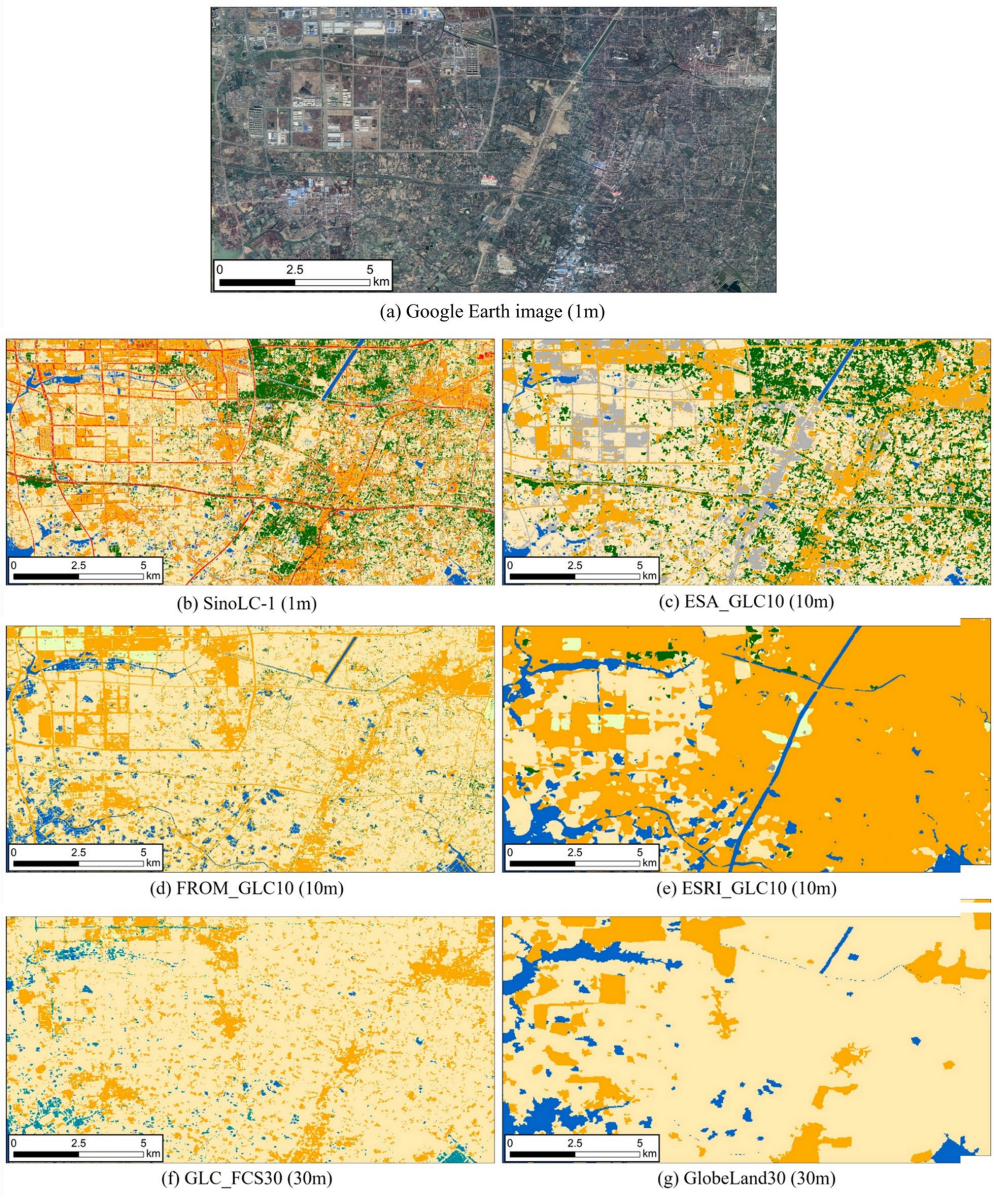
Demonstration of the visual comparison for Changzhou City, Jiangsu Province. The VHR remote sensing image in the figure is from Google Earth 2021.

Figure 2 demonstrates the performance of SinoLC-1 at a larger spatial scale in Changzhou City, Jiangsu Province, compared to other LULC products at different resolutions. Analyzing the VHR satellite image in Fig. 2a, it can be observed that the land cover representation in ESRI_GLC10 in Fig. 2e and GlobeLand30 in Fig. 2g is the most ambiguous, with significant confusion between agricultural land, buildings, and forests in urban areas. GLC_FCS30 exhibits the poorest performance in terms of forest cover, transportation roads, and rivers. FROM_GLC10 shows accurate performance in representing water bodies such as artificial lakes and rivers, but its performance in forest cover types falls short of expectations. ESA_GLC10 performs relatively well compared to other contrasting products, but still shows deficiencies in representing water bodies. In comparison to these GLC products, SinoLC-1 demonstrates the best overall performance, exhibiting fine details of land cover such as small rivers, artificial lakes, ponds, vegetation, and buildings. It accurately identifies boundaries between different LULC types, which contributes to the fine modeling of the research area using the SWAT model.

### Soil and water assessment tool (SWAT)

SWAT was developed by Dr. Jeff Arnold at the Agricultural Research Center of the United States Department of Agriculture (USDA) in 1994. It was designed to assess the impacts of land management, vegetation changes, groundwater extraction, and reservoir management on water quality and quantity. It can be traced back to the USDA-ARS early field-scale models: the Groundwater Loading Effects of Agricultural Management Systems (GLEAMS) model, the Chemical, Runoff, and Erosion from Agricultural Management Systems (CREAMS) model, and the Environmental Policy Integrated Climate (EPIC) model. It simulates evapotranspiration, water movement in the soil, lateral surface runoff, and groundwater flow based on several empirical equations and the principle of water balance. The modeling process of SWAT can be divided into the surface and routing components. A watershed is divided into several sub-basins, which can further be divided into Hydrologic Response Units (HRUs). Each HRU has the same LULC type, soil type, watershed management, and hydrological processes. In the surface component, the Soil Conservation Service (SCS) method is used to independently calculate infiltration and surface runoff in each HRU, and the water yield is computed at the outlet of the sub-basin. Subsequently, the routing process, including flood routing and sediment transport, is performed using simulation computations. The model has a strong physical foundation and can simulate hydrological processes at different spatial and temporal scales. It is capable of generating weather data using weather generators in areas with no observational data. The model has been widely applied in many countries and regions^39^.

The river network was generated from the DEM through repeated debugging. By setting a minimum contributing area of 500 km^2^ and threshold values of LULC, soil type, and slope as 13%, 20%, and 20% respectively, the hydrological characteristics of the study area could be accurately characterized. Eventually, 275 sub-basins and 2571 Hydrological Response Units (HRUs) were generated. HRUs serve as the smallest simulation units in the SWAT model, with identical topographic slope, LULC, and soil type within the same HRU. The SWAT model was calibrated separately for each controlled sub-basin from January 1, 2015, to December 31, 2017, and validated from January 1, 2018, to December 31, 2022.

Following previous research in HRB^40^ and other SWAT studies in neighboring regions^41^, parameters with a P-value less than 0.4 were selected for calibration in the sub-basins controlled by the hydrological stations. SWAT-CUP is an open-source program used for calibrating the SWAT model. This program incorporates five algorithms into the SWAT model, with the SUFI-2 algorithm being found highly effective in large-scale model calibration^42^. Therefore, we utilized the SUFI-2 algorithm for sensitivity analysis and parameter optimization of the hydrological model^43^.

### Bidirectional long short-term memory (BiLSTM) model

**Figure 3:**
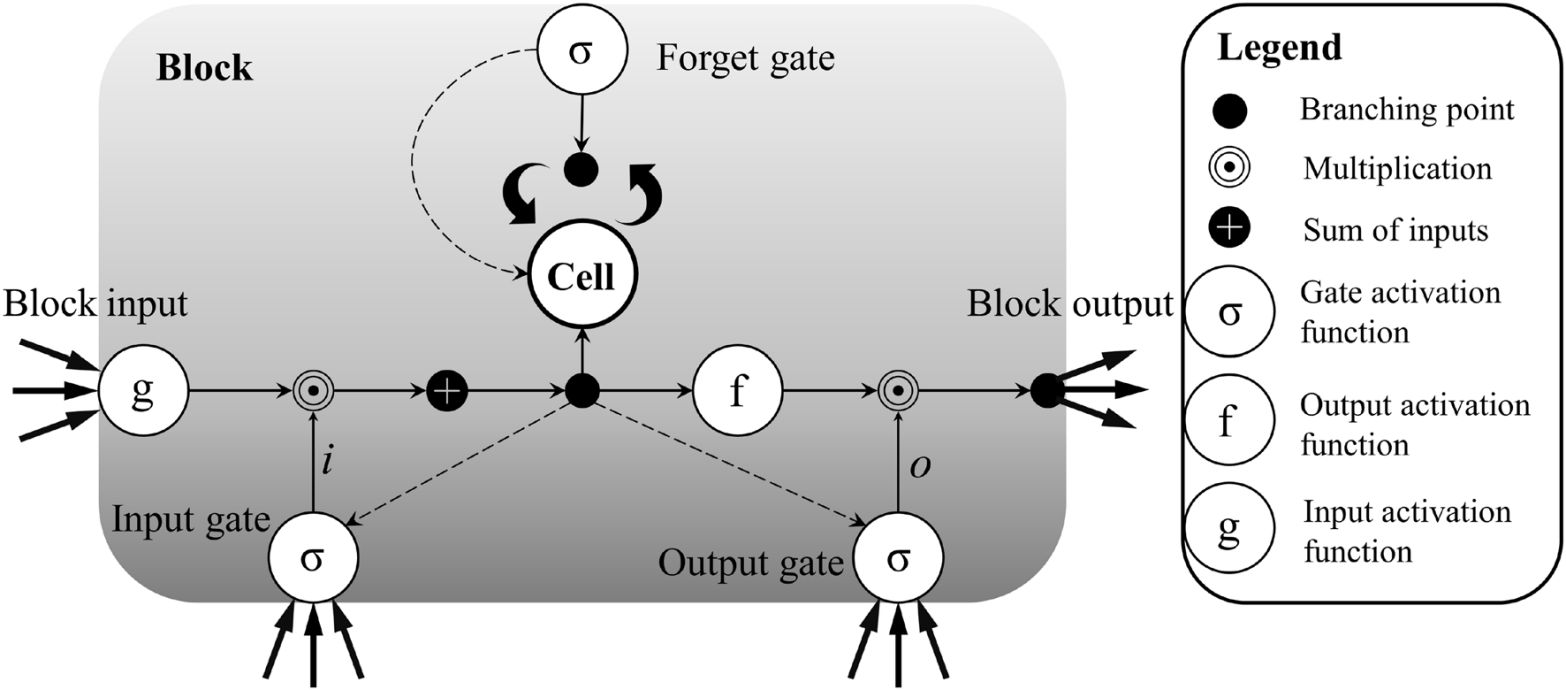
Illustration of an LSTM block.

In various deep learning neural networks, recurrent neural networks (RNNs) have been widely applied to process various time series data^44^. However, when traditional RNNs are used to handle long time series data, issues such as gradient explosion or vanishing gradients may arise. Therefore, the LSTM neural network was proposed to address these problems encountered by traditional RNNs^27,35^. In LSTM, memory cells containing input gates, forget gates, and output gates are used instead of hidden functions in RNNs (Fig. 3). The memory cells can make decisions to retain or forget the memory from the previous time step, enabling the preservation of information for long sequences. The mechanisms of the three gates are represented by the following formulas:

Forget gate:

1 $$f_{t} = \sigma \left( {W_{f} \cdot \left[ {h_{t - 1} ,x_{t} } \right] + b_{f} } \right)$$

Input gate:

2 $$i_{t} = \sigma \left( {W_{i} \cdot \left[ {h_{t - 1} ,x_{t} } \right] + b_{i} } \right)$$

3 $$\tilde{C}_{t} = \tanh \left( {W_{C} \cdot \left[ {h_{t - 1} ,x_{t} } \right] + b_{C} } \right)$$

4 $$C_{t} = f_{t} \odot C_{t - 1} + i_{t} \odot \tilde{C}_{t}$$

Output gate:

5 $$o_{t} = \sigma \left( {W_{O} \cdot \left[ {h_{t - 1} ,x_{t} } \right] + b_{O} } \right)$$

6 $$h_{t} = O_{t} \odot \tanh \left( {C_{t} } \right)$$

Here, ${f}_{t}$, ${i}_{t}$, and ${o}_{t}$ represent the forget gate, input gate, and output gate, respectively. ${C}_{t}$ is the cell memory, ${h}_{t}$ is the output of the neuron's short-term memory at time $t$, ${\widetilde{C}}_{t}$ is the new input to the memory, $h$ is the hidden vector, $\sigma$ is the activation function, $W$ is the weight matrix, $b$ is the bias term, [M, N] denotes the concatenation of two vectors, and ⨀ represents element-wise multiplication.

The BiLSTM neural network model consists of two stacked LSTM models in opposite directions. Compared to the traditional LSTM neural network, the BiLSTM incorporates a bidirectional recurrent neural network that includes both forward and backward propagation. This overcomes the deficiency of insufficient information mining in the unidirectional LSTM neural network, allowing for effective capture of the correlation information between explanatory and target variables.

**Figure 4:**
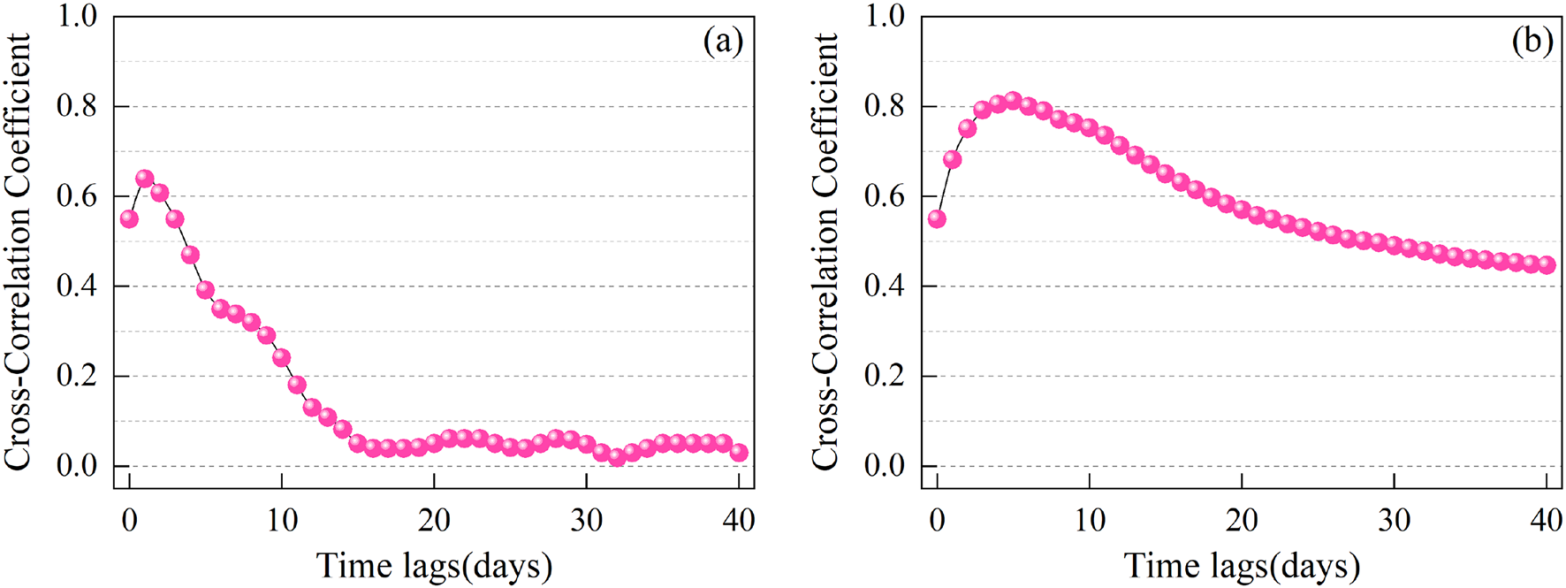
Correlation analysis between daily streamflow and (**a**) daily rainfall; and (**b**) total rainfall.

Previous studies^45,46^ have demonstrated a strong correlation between daily streamflow and rainfall in nearby areas over a short-term period. In this study, we conducted a correlation analysis between the streamflow of the six major rivers in the HRB (Zhangwei River, Ziya River, Daqing River, Yongding River, Beiyun River, and Hai River) and the total rainfall in the adjacent areas over the preceding n days, as well as the daily rainfall (Fig. 4). The results indicate that the cumulative rainfall over the previous six days (P_6_) exhibits the highest correlation with daily streamflow compared to rainfall on individual days. Considering the flood generation process in the HRB, which can be divided into two stages, the first stage involves rainfall that saturates the soil, followed by the second stage where rainfall leads to widespread runoff throughout the watershed, quickly flowing into the rivers and causing a rapid rise in water levels. Finally, we selected the P_6_ as the covariate in the neural network model to enhance the performance of the coupled model.

To improve the performance of BiLSTM in simulating daily flow, it is essential to configure the appropriate network architecture and hyperparameters. To determine the optimal combination of hyperparameters for the BiLSTM model, we modeled the hyperparameters using Gaussian processes. We employed an improved Markov chain Monte Carlo (IMCMC) algorithm to accelerate the training of the Gaussian process model. Specifically, the IMCMC algorithm was used to compute the hyperparameters of the Gaussian surrogate model, such as length scales and covariance amplitudes. Additionally, we utilized an improved Thompson sampling (ITS) method as the acquisition function to obtain the next sampling point and compute the corresponding loss value, which was then incorporated into the historical observation set. This iterative process continued until a set of well-performing hyperparameters was obtained^47,48^. To mitigate overfitting, an early stopping strategy^49^ is implemented. This strategy halts the training process as soon as the model's performance on the validation set starts to decline.

The loss function of the BiLSTM model is used to measure the difference between the predicted values and the true values. It serves as the metric for the optimization algorithm to adjust the model's parameters in order to minimize the error between the predicted values and the true values. By defining an appropriate loss function, the model can be guided to learn the correct patterns and rules, thereby improving its performance and accuracy. In this study, since the BiLSTM model is addressing a regression problem, the weighted sum of the two mentioned loss functions is chosen as the loss function, as shown below:

7 $$L_{MAE} = \frac{1}{n}\Sigma_{{{\text{i}} = 1}}^{n} \left| {\hat{y}_{i} - y_{i} } \right|$$

8 $$L_{RMSE} = \sqrt {\frac{{\Sigma_{{{\text{i}} = 1}}^{n} \left( {\hat{y}_{i} - y_{i} } \right)^{2} }}{n}}$$

9 $$Loss_{sum} = weight_{MAE} *L_{MAE} + weight_{RMSE} *L_{RMSE}$$

Here, ${\widehat{y}}_{i}$ represents the predicted streamflow values given by the BiLSTM model based on the quick flow and base flow, while ${y}_{i}$ represents the observed streamflow values. $weigh{t}_{MAE}$ stands for the weight of the Mean Absolute Error loss (${L}_{MAE}$), and $weigh{t}_{RMSE}$ stands for the weight of the Root Mean Square Error loss (${L}_{RMSE}$).

After conducting multiple experiments, the optimal configuration for the BiLSTM model was determined as follows: The first hidden layer consists of 512 neurons, followed by four dense layers with 256, 64, 32, and 1 neuron, respectively. Rectified Linear Unit (ReLU) is a commonly used activation function in deep learning that is widely employed. Compared to other activation functions such as the sigmoid function and tanh function, ReLU offers faster computation and better handling of gradient sparsity and nonlinearity. To alleviate the issue of vanishing gradients, ReLU activation function is utilized in the hidden layers^50^.

### Coupling SWAT with BiLSTM

To explore the parameter settings that lead to optimal performance of the model, we conducted a comparative analysis between two sets of hybrid models. First is the SWAT-D-BiLSTM, where "D" denotes that all parameters in SWAT are set to their default values. However, the structure, equations, and databases of the SWAT model were developed based on relevant research conducted by the US Department of Agriculture. The default parameter values primarily reflect the soil properties and climatic conditions of the United States, while HRB has a vast geographical area and diverse watershed characteristics. Using default parameter values may not adequately simulate the actual climate conditions and properties of the HRB. This means that using an uncalibrated SWAT model directly as a transfer function from rainfall to runoff could introduce systematic errors. Therefore, in SWAT-C-BiLSTM, we calibrated some of the parameters in SWAT. However, the SWAT model has numerous parameters, and acquiring data for all parameters is time-consuming and challenging, with some data being difficult to obtain accurately due to practical limitations. Furthermore, the calibration process of the SWAT model involves the intricate issue of equifinality, and calibrating a large number of parameters introduces significant uncertainty^51^. Based on these considerations, in order to reduce model uncertainty and minimize unnecessary time and effort while ensuring model simulation performance, we conducted a sensitivity analysis using SWAT-CUP for the sub-basins controlled by 21 hydrological stations. In sensitivity analysis, the *P*-value reflects the significance level of parameter sensitivity, with values closer to 0 indicating higher parameter sensitivity. Based on previous relevant studies conducted in the HRB and neighboring regions, we selected all parameters with P-values less than 0.4 for calibration in the SWAT-C-BiLSTM model^52^. Table 2 presents the sensitivity parameter rankings and calibrated parameter values for nine key hydrological stations.

**Table 2:** Calibrated SWAT parameters in SWAT-C-BiLSTM.

Station	Parameter	*P*-value	Calibrated valuer
Chengde	CN2	0	0.105
	CANMX	0.085	0.091
	SOL_Z	0.128	−0.264
	CH_N1	0.16	0.183
	SOL_AWC	0.217	0.105
	SOL_BD	0.226	−0.045
	CH_N2	0.227	0.181
	GWQMN	0.29	2375
Sandaohezi	CN2	0	−0.609
	SOL_BD	0.026	0.035
	CANMX	0.042	0.497
	SOL_Z	0.071	−0.664
	CH_K2	0.157	37.491
	CH_N2	0.192	0.085
	RCHRG_DP	0.2	0.275
	ALPHA_BNK	0.208	0.705
	CH_N1	0.24	0.207
	HRU_SLP	0.269	0.154
	SOL_AWC	0.329	−0.055
	ESCO	0.388	−0.190
Xiabao	CN2	0	0.061
	SLSUBBSN	0.002	0.149
	SOL_K	0.01	0.006
	ALPHA_BF	0.068	0.994
	ALPHA_BNK	0.109	0.659
	REVAPMN	0.125	44.375
	SOL_Z	0.238	0.126
	SOL_BD	0.332	0.339
	ESCO	0.336	0
Jiuwangzhuang	CH_K2	0	35.759
	RCHRG_DP	0	0.071
	SOL_K	0.005	0.161
	REVAPMN	0.025	87.884
	SOL_Z	0.035	−0.378
	OV_N	0.13	0.030
	GW_REVAP	0.21	0.020
	CH_N2	0.225	0.150
	GWQMN	0.36	4091.569
Pingshan	CN2	0.002	−0.191
	SOL_AWC	0.0285	−0.500
	SOL_BD	0.0292	0.316
	CH_N1	0.0572	−0.289
	CH_K1	0.0594	−0.371
	ESCO	0.0872	0.000
	SOL_Z	0.195	0.551
	SOL_K	0.219	0.272
	GWQMN	0.239	3897.165
	GW_REVAP	0.291	0.160
Aixinzhuang	CN2	0	−0.308
	ALPHA_BNK	0.007	0.741
	SLSUBBSN	0.035	0.208
	ALPHA_BF	0.061	0.292
	SOL_K	0.074	0.387
	SOL_BD	0.107	−0.125
	GW_DELAY	0.286	159.500
	SLSOIL	0.297	0.700
	REVAPMN	0.349	357.643
	CH_N1	0.372	0.042
Zhangfang	CN2	0	0.199
	SOL_BD	0	−0.259
	CH_K2	0.013	305.535
	ESCO	0.014	0.418
	SOL_Z	0.016	0.059
	GWQMN	0.034	2718.765
	RCHRG_DP	0.045	0.942
	SOL_K	0.147	−0.412
	EPCO	0.161	0.200
	OV_N	0.232	0.0234
	SLSUBBSN	0.248	0.557
	GW_REVAP	0.293	0.020
	HRU_SLP	0.353	−0.166
	ALPHA_BNK	0.367	0.417

**Figure 5:**
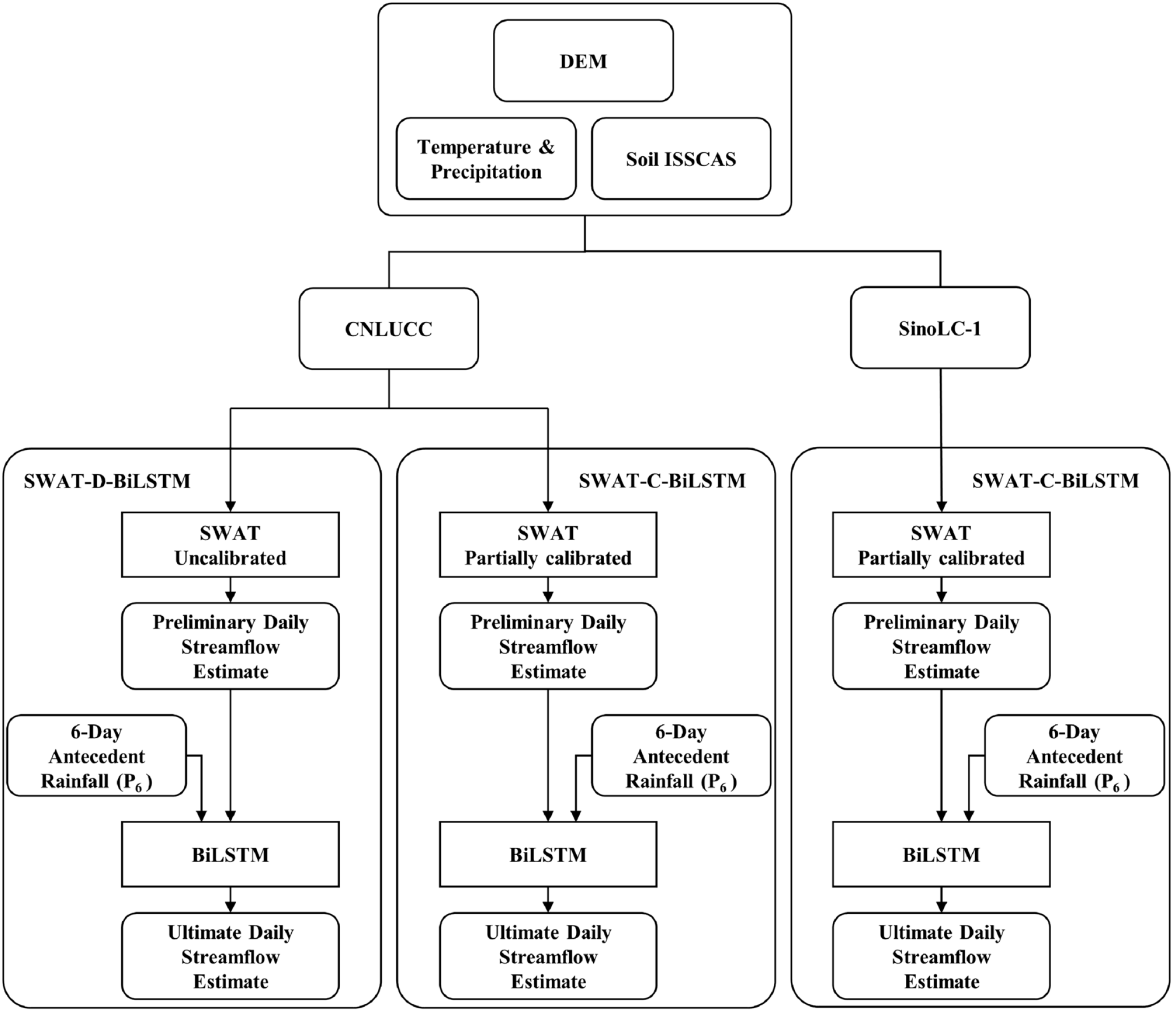
Configuration of the three hybrid models.

The daily streamflow estimates generated by the uncalibrated SWAT model and the partially calibrated SWAT model were input into two identical BiLSTM models. Additionally, in both cases, the total precipitation of the previous six days (P_6_) was used as a covariate for BiLSTM (Fig. 5). The BiLSTM component in both hybrid models shared the same structure as the standalone BiLSTM model. In general, this study conducts a comparative analysis of the performance of five models, including two standalone models (BiLSTM and uncalibrated SWAT) using CNLULC, and three hybrid models (uncalibrated SWAT-D-BiLSTM, partially calibrated SWAT-C-BiLSTM using CNLULC, and partially calibrated SWAT-C-BiLSTM with SinoLC-1).

### Model performance evaluation

In this study, five metrics were used to compare the performance of different models and assess the impact of VRH data on the simulation results. Regarding the selection of LUCC data, except for SWAT-C-BiLSTM with SinoLC-1, other independent or hybrid models utilized CNLUCC. The five metrics and their respective calculation formulas are as follows:

10 $$NSE = 1 - \frac{{\mathop \sum \nolimits_{i = 1}^{N} \left( {M_{i} - S_{i} } \right)^{2} }}{{\Sigma_{{{\text{i}} = 1}}^{N} \left( {M_{i} - \overline{M}_{i} } \right)^{2} }}$$

11 $$R^{2} = 1 - \frac{{\Sigma_{i = 1}^{N} \left( {M_{i} - S_{i} } \right)^{2} }}{{\Sigma_{i = 1}^{N} \left( {M_{i} - \overline{M}_{i} } \right)^{2} \Sigma_{{{\text{i}} = 1}}^{N} \left( {S_{i} - \overline{S}_{i} } \right)^{2} }}$$

12 $$RMSE = \sqrt {\frac{{\Sigma_{{{\text{i}} = 1}}^{n} \left( {S_{i} - M_{i} } \right)^{2} }}{n}}$$

13 $$RSR = \frac{{\sqrt {\mathop \sum \nolimits_{i = 1}^{n} \left( {S_{{\dot{i}}} - M_{i} } \right)^{2} } }}{{\sqrt {\mathop \sum \nolimits_{i = 1}^{n} \left( {M_{{\dot{i}}} - \overline{M}_{i} } \right)^{2} } }}$$

14 $$PBIAS = \frac{{\mathop \sum \nolimits_{i = 1}^{n} 100\left( {M_{i} - S_{i} } \right)}}{{\mathop \sum \nolimits_{i = 1}^{n} M_{i} }}$$

Here, ${M}_{i}$ and ${S}_{i}$ represent the measured and simulated values, respectively, and ${\overline{M} }_{i}$ and ${\overline{S} }_{i}$ represent the mean of measured values and the mean of simulated values, respectively.

Among the five metrics mentioned above, NSE (Nash–Sutcliffe Efficiency) is used to assess the consistency between model predictions and observed data, with a range of values from negative infinity to 1. A value closer to 1 indicates better predictive ability of the model. R^2^ (Coefficient of Determination) is a measure of model fit, indicating the model's ability to explain the variability of the dependent variable. It ranges from 0 to 1, and a value closer to 1 indicates better explanatory power of the model for the observed data. RMSE (Root Mean Square Error) represents the root mean squared error between predicted and observed values, where a smaller RMSE indicates better predictive ability of the model. RSR is the ratio of RMSE to the standard deviation of observed data, and a value closer to 0 indicates more accurate model predictions. PBIAS (Percent Bias) represents the percentage deviation of the model's predicted values from the observed values. Negative values indicate underestimation, while positive values indicate overestimation by the model. PBIAS helps assess the overall prediction bias of the model for the observed data.

### Assessment of climate change impact on streamflow

CMIP6 (Coupled Model Intercomparison Project Phase 6) is an international collaborative project aimed at evaluating the performance and predictive capabilities of global climate models under different climate change scenarios^53^. ScenarioMIP (Scenario Model Intercomparison Project) is a sub-project of CMIP6 that aims to compare and assess the performance of global climate models under different socio-economic scenarios^54^. ScenarioMIP provides researchers with a series of simulated experiments and datasets to explore climate change trends and possibilities under different socio-economic scenarios. These scenarios involve variations in factors such as population growth, economic development, energy use, and greenhouse gas emissions.By comparing the simulation results under different scenarios, ScenarioMIP can provide more detailed and comprehensive information about the possibilities of future climate change. This information serves as a scientific basis for formulating climate policies and sustainable development strategies. Among these scenarios, SSP1-2.6 represents a combination of low societal vulnerability, low mitigation pressure, and low radiative forcing, which is an updated version of the RCP2.6 scenario from CMIP5. SSP2-4.5 represents a combination of moderate societal vulnerability and moderate radiative forcing, also an updated version of the RCP4.5 scenario from CMIP5^55^. SSP3-7.0 is a newly added radiative forcing scenario in CMIP6, representing a combination of high societal vulnerability and relatively high anthropogenic radiative forcing. SSP5-8.5 is an updated version of the RCP8.5 scenario from CMIP5, which is the only scenario that achieves a shared socioeconomic pathway with anthropogenic radiative forcing reaching 8.5 W/m^2^ by 2100^56^.

Table 3 shows the five CMIP6 models used in this study and the resolution of the temperature and precipitation forecasts they provide. Previous studies have demonstrated the satisfactory performance of these models in the HRB^57,58^. In this study, the predictions of GCMs under three emission scenarios (SSP1-2.6, SSP2-4.5 and SSP5-8.5) were employed.

**Table 3:** Five CMIP6 models used in this study.

Model	Institution	Abbreviation	Latitude × Longitude
IPSL-CM6A-LR	Institut Pierre-Simon Laplace, France	IPSL	2.5° × 1.2676°
FGOALS-g3	Institute of Atmosphere Physics, Chinese Academy of Science, China	FGO	2.0° × 2.0°
MIROC6	Japan Agency for Marine-Earth Science and Technology, Japan	MIR	1.6063° × 1.4°
CanESM5	The Canadian Centre for Climate Modelling and Analysis, Canada	CAN	2.8° × 2.8°
MRI-ESM2-0	Max-Planck-Institute for Meteorology, Germany	MRI	1.125° × 1.12°

To assess the regional impacts of climate change, predictions from global climate models must undergo downscaling due to their coarse resolution. One statistical downscaling method used for this purpose is weather generators. The LarsWG weather generator was developed by Lausanne Laboratory to promote the development of a crop growth model and hydrological model. The LarsWG weather generator can generate a series of meteorological data for a certain period in the future based on historical meteorological data and geographical data, such as rainfall, SR, T_max_, and T_min_. The generator has been widely applied in the projections of crop yield, soil erosion, and extreme environmental events^59,60^. In this study, we utilized LarsWG to downscale the projections from five GCMs in the CMIP6 dataset across three emission scenarios, producing daily rainfall and temperature data. Due to the potential variations in outcomes when utilizing different models for simulation and prediction, in order to mitigate random errors and model uncertainties, we computed the ensemble mean of projections from five GCMs under the same emission scenarios, denoted as "Ens" in the results. When calculating the ensemble mean, it's necessary to first calculate the average data for each individual model over a specific period at corresponding locations (spatial grid points). Finally, the average values from all models are averaged once again. Employing the ensemble mean helps to reduce errors arising from the stochastic fluctuations of individual models, providing more stable and reliable predictions and analytical results.

Daily precipitation and temperature records from the Zhangjiakou weather station for a total of twenty years from 2002 to 2022 were used to calibrate the LarsWG, which was then run to downscale the monthly projections for the five GCMs under different emission scenarios. Daily projections were generated for a total of forty years for the mid-century (2040–2060) and late century (2080–2100) under different emission scenarios. The predicted data were then fed into the best performing model to assess the impact of climate change on streamflow within the HRB.

## Results and discussion

### Streamflow simulation performance comparison

The accuracy of the daily streamflow simulation of the five models based on the five metrics at different hydrological stations is presented in Table 4 in the form of mean values. It is evident that SWAT-C-BiLSTM with SinoLC-1 consistently exhibits the highest accuracy across both simulation periods. During the calibration period, it achieves NSE of 0.93 and R^2^ of 0.95, while in the validation period, the values remain high at 0.92. In contrast, when using CNLUCC data, SWAT-C-BiLSTM shows slightly lower accuracy with NSE and R^2^ of 0.90 and 0.92 during the calibration period, decreasing to below 0.90 during the validation period. These results highlight the significant effect of utilizing SinoLC-1 data in enhancing simulation performance. Furthermore, a comparison between the two hybrid models using CNLUCC data reveals that the hybrid model employing default SWAT parameters performs noticeably worse compared to the hybrid model with partially calibrated SWAT parameters across both simulation periods. During the calibration period, the NSE and R^2^ for the hybrid model with default parameters are 0.82 and 0.85, both falling below 0.90, and further declining to below 0.80 during the validation period. These findings emphasize the effectiveness of the strategy involving partial calibration of SWAT parameters. The two independent models consistently exhibit the lowest accuracy throughout the simulation periods, particularly the independent BiLSTM model, which had NSE and R^2^ of 0.51 and 0.59 respectively, both below 0.60 in the validation period when simulations were performed using non-training data.

**Table 4:** Performance comparison.

Model	Calibration period (2015–2017)					Validation period (2018–2022)				
	NSE	R^2^	RMSE (m^3^/s)	RSR	PBIAS (%)	NSE	R^2^	RMSE (m^3^/s)	RSR	PBIAS (%)
SWAT	0.81	0.83	223.7	0.46	24.1	0.76	0.85	417.3	0.49	10.83
BiLSTM	0.71	0.73	294.1	0.51	−11.4	0.51	0.59	589.4	0.73	−29.21
SWAT-D-BiLSTM	0.82	0.85	160.0	0.39	6.49	0.77	0.80	478.1	0.55	−16.9
SWAT-C-BiLSTM	0.90	0.92	147.2	0.33	1.12	0.86	0.89	265.9	0.39	−4.79
SWAT-C-BiLSTM with SinoLC-1	0.93	0.95	126.3	0.27	−0.91	0.92	0.92	221.0	0.34	−1.02

In addition, the RMSE and RSR of SWAT-C-BiLSTM with SinoLC-1 were consistently the lowest among the five models at both periods, with values of 221.0 and 0.34 respectively during the validation period. The RMSE during the validation period for SWAT-C-BiLSTM with SinoLC-1 was lower than the other four models by 47.0%, 62.5%, 53.8%, and 16.9% respectively. It is worth noting that the PBIAS of SWAT-C-BiLSTM with SinoLC-1 during the calibration period was -0.91% and -1.02% during the validation period, both of which remained at the lowest levels. This indicates that the model did not show significant systematic bias. In contrast, the independent BiLSTM model had a PBIAS during the calibration period lower than the independent SWAT model, but it reached -29.21% during the validation period. This indicates that the model exhibited significant systematic bias during the validation period, severely underestimating the overall streamflow of HRB. These findings are consistent with prior research. BiLSTM demonstrates favorable predictive performance during the calibration period, but its model performance significantly deteriorates when the simulation period falls outside the scope of the training data, due to the insufficient representativeness of the training data^23^. On the other hand, the SWAT model exhibits a trend of insufficient peak flow prediction, stemming from inherent limitations within the SWAT model itself^18,19^. The coupled model addresses both of these issues, enhancing peak flow prediction accuracy, and simultaneously showcasing outstanding performance during the validation period.

**Figure 6:**
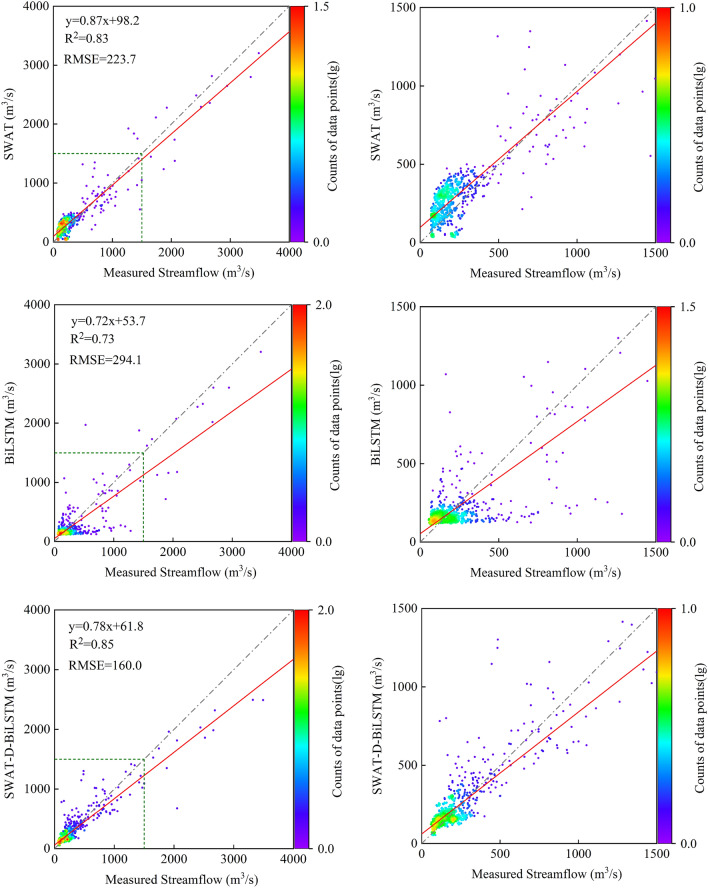

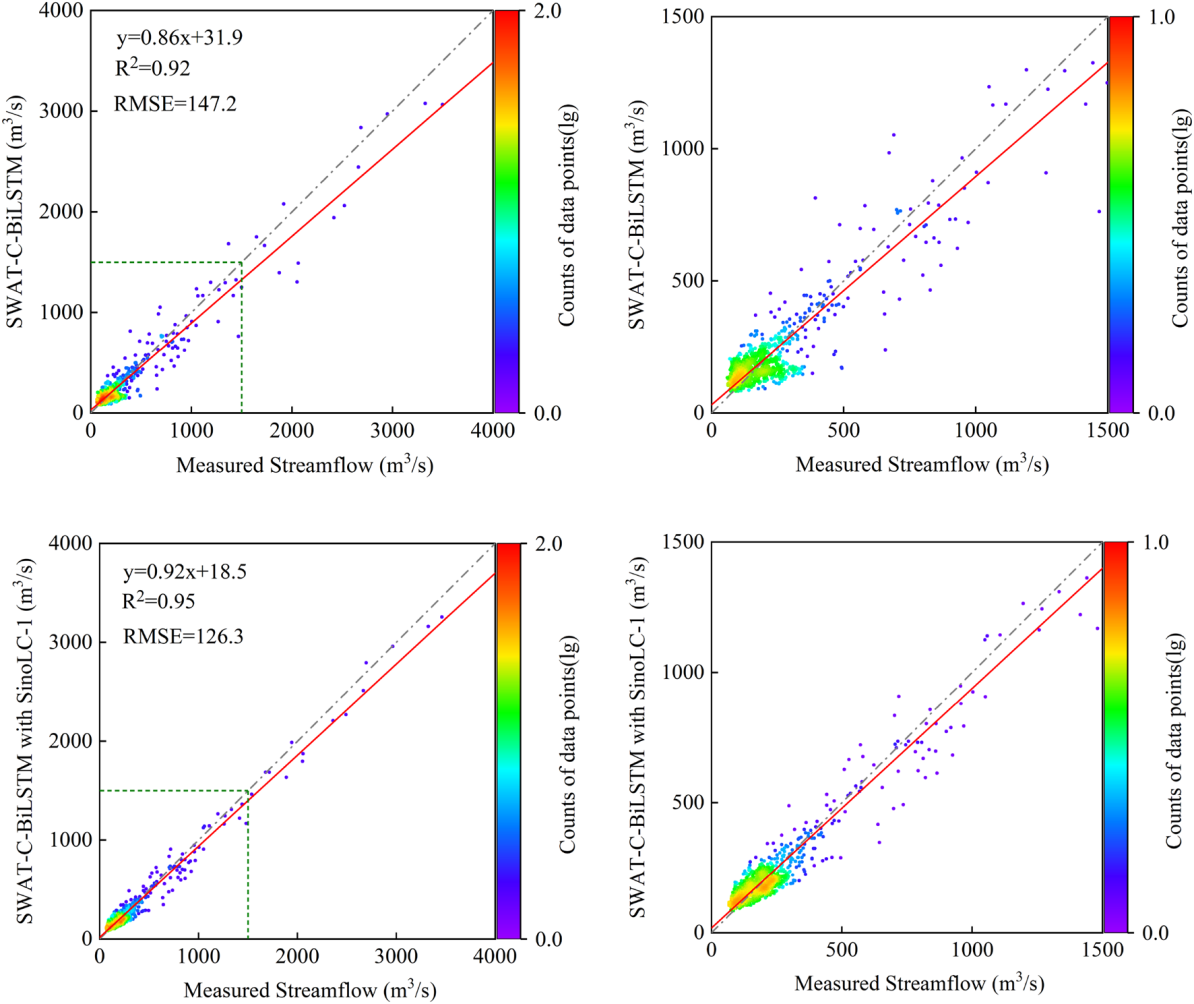
Scatterplots between measured and simulated daily streamflow during the calibration period.

**Figure 7:**
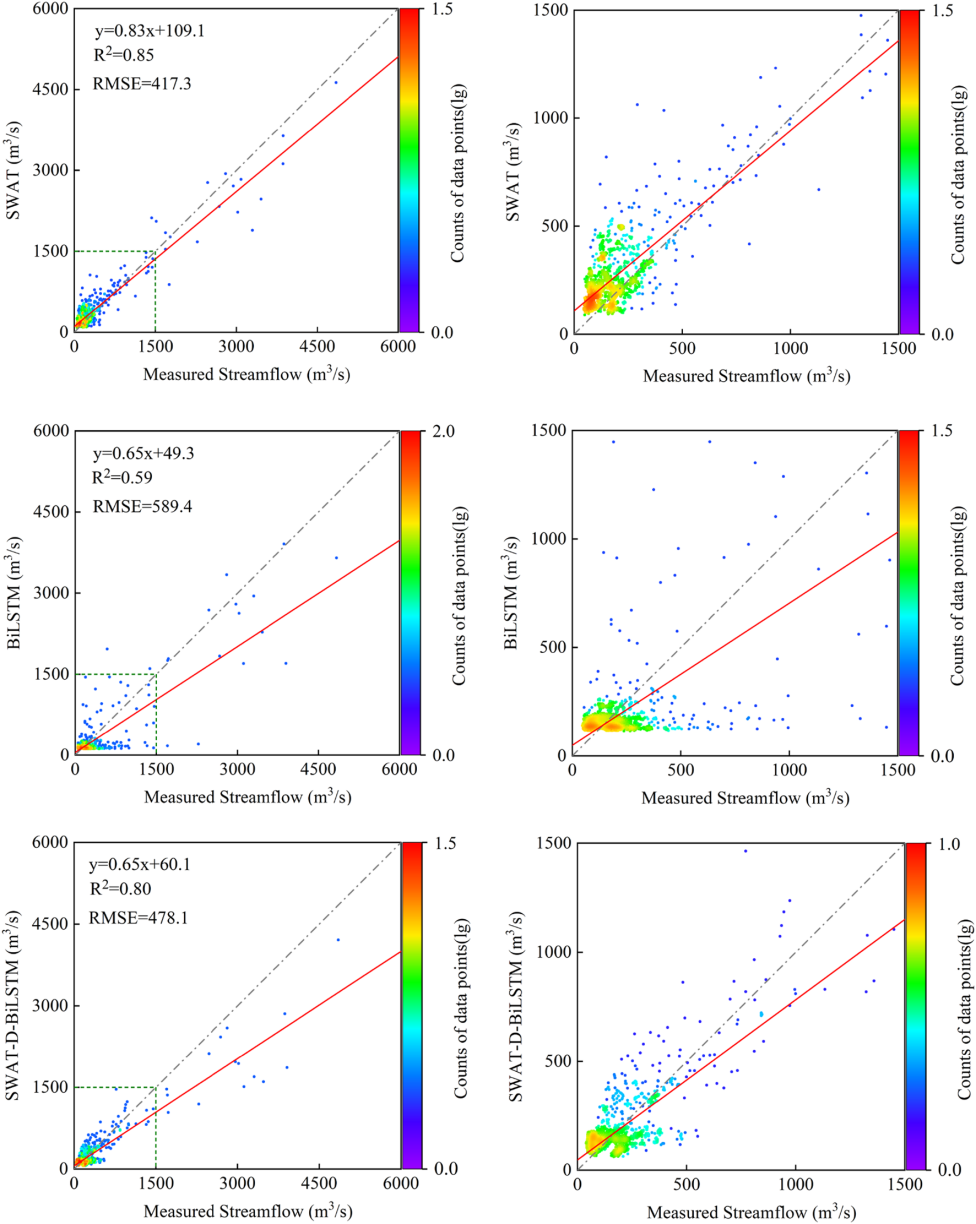

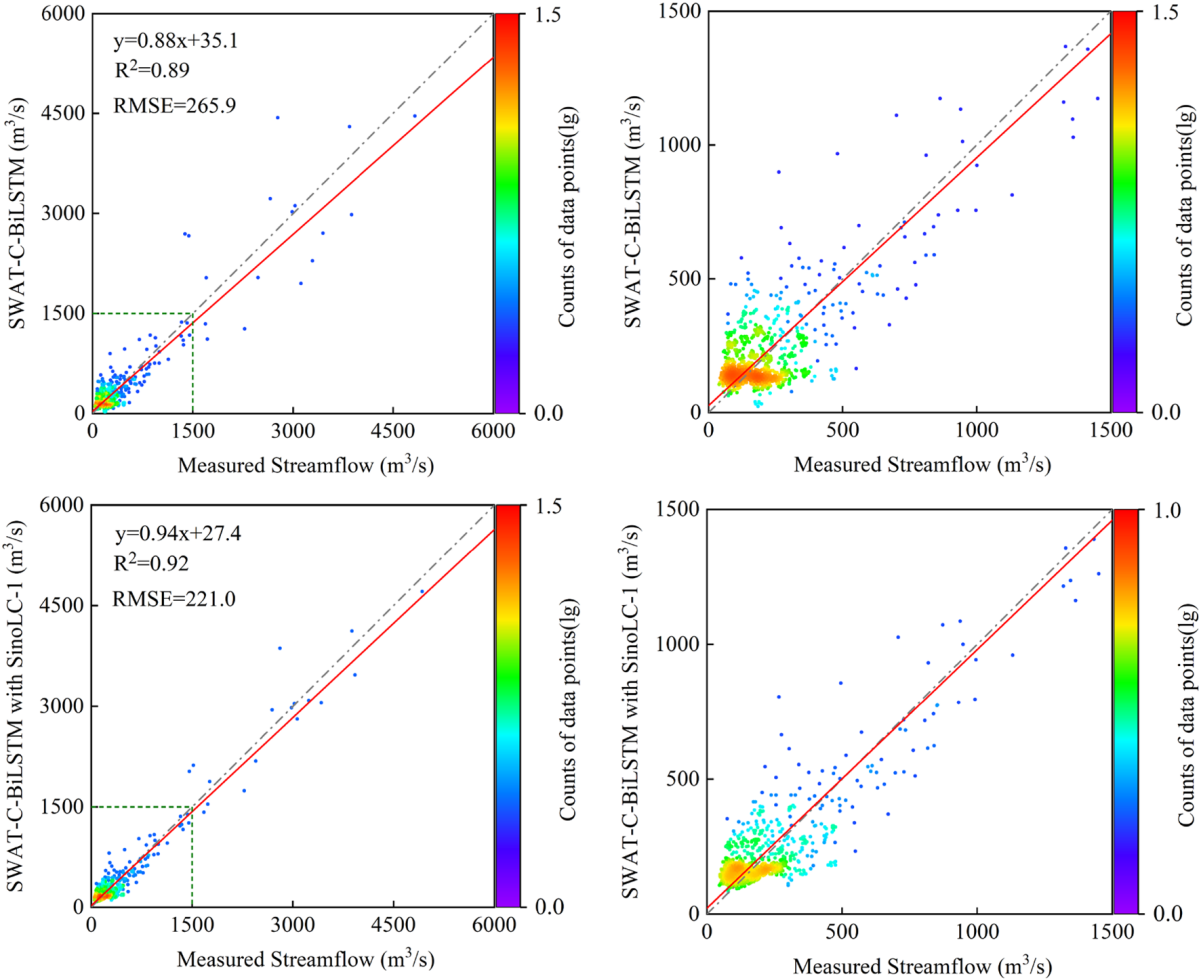
Scatterplots between measured and simulated daily streamflow during the validation period.

Figures 6 and 7 present the scatter plots of daily streamflow data for all models during the calibration and validation periods, with the right side magnifying the scatter distribution below 1500 m^3^/s. The figures also show the results of linear regression analysis between simulated values and measured values. During the calibration period, although all models significantly underestimated streamflow above 1500 m^3^/s, the simulated values generated by SWAT-C-BiLSTM with SinoLC-1 were overall closest to the measured values. Looking at the degree of deviation from the regression line, the regression coefficient of the independent BiLSTM model was 0.72, which differed the most from 1. This indicates that the performance of this model was the poorest. It is noticeable that for the scatter distribution of streamflow below 500m^3^/s, the independent SWAT model showed a noticeable difference from the other four models, with a clear clustering of points above the Y = X line. This suggests that compared to the other models, this model significantly overestimated streamflow below 500m^3^/s as a whole.

During the validation period, SWAT-C-BiLSTM with SinoLC-1 had the best simulation performance for both the streamflow below 1500 m^3^/s and the peak flows among all models, with a regression coefficient of 0.94, closest to 1. In contrast, SWAT-D-BiLSTM had a regression coefficient of 0.65, the same as the independent BiLSTM model, showing the largest deviation from 1. The independent SWAT model had a regression coefficient of 0.83, indicating that coupling an uncalibrated SWAT model with BiLSTM led to a significant decrease in the accuracy of the prediction results.

As a purely data-driven model, the modeling performance of BiLSTM is greatly influenced by the input data used for training. If the representativeness of the input data is poor, the performance of BiLSTM may not meet expectations. The most common method for selecting training data is to randomly sample the complete observed time series to ensure that the samples reflect the overall temporal variations throughout the study period. In this study, the BiLSTM model was trained solely using flow measurements from the calibration period. However, the validation period (2018–2022) experienced more pronounced flood events compared to the calibration period. The lack of training samples representing the highest flow events could potentially contribute to the relatively poorer performance of the BiLSTM model in simulating daily streamflow during the validation period. On the other hand, the SWAT model, developed by the United States Department of Agriculture, incorporates default parameter values that are extensively researched and tailored to local physical and climatic conditions. However, it should be noted that the HRB, with its vast geographical extent and diverse watershed characteristics, differs significantly from the regions in the United States for which the SWAT model was originally developed. Using default parameter values as transfer functions inevitably leads to significant systemic biases, resulting in relatively poorer performance of SWAT-D-BiLSTM. However, by calibrating only the parameters with a P-value less than 0.4, the SWAT-C-BiLSTM achieved a significant improvement in daily streamflow simulation performance while maintaining a low uncertainty level.SWAT-C-BiLSTM with SinoLC-1 utilizes VRH data with a resolution of 1 m. Compared to SWAT-C-BiLSTM, it can accurately identify complex and overlapping land types, significantly reducing the confusion between different LULC types. For example, in regions where farmland, urban areas, and forests intersect, it correctly distinguishes between farmland and urban areas. Additionally, at a 30-m resolution data (CNLUCC), similar LULC types are merged into the same category, such as cropland and paddy fields being classified as paddy fields. These confusions or mergers affect the subsequent HRU delineation. During HRU delineation, as the threshold increases, the area of merged LULC types also increases, resulting in a decrease in simulation accuracy. Therefore, overall, the estimates produced by SWAT-C-BiLSTM with SinoLC-1 are slightly better than those of SWAT-C-BiLSTM.

### Climate change impact assessment

#### Projected changes in rainfall and temperature

**Figure 8:**
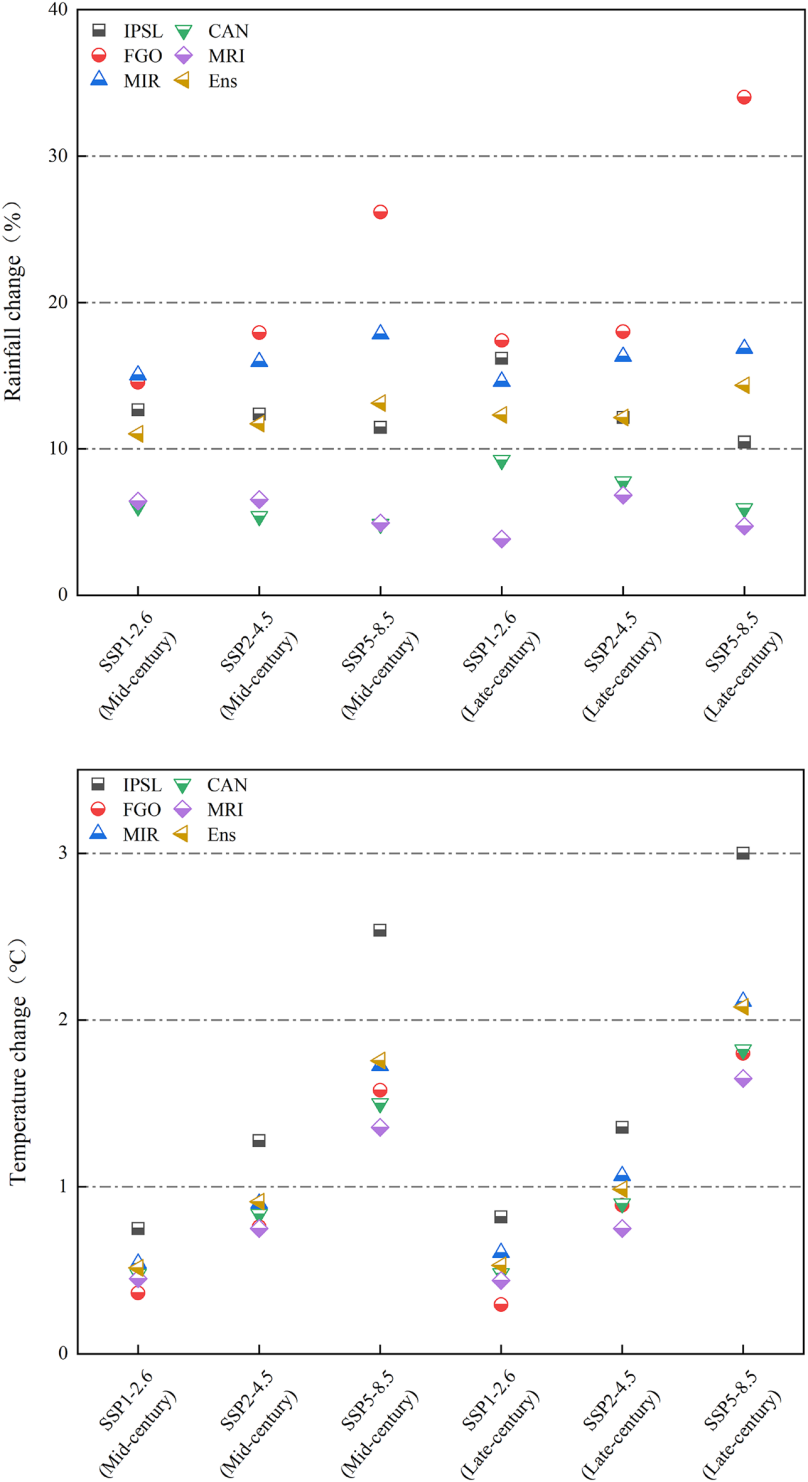
Relative change in annual mean rainfall and absolute change in annual mean temperature.

Figure 8 provides visual representation of the projected relative changes in average rainfall and absolute changes in average temperature for the mid-century (2040–2060) and late-century (2080–2100) across three emission scenarios (SSP1-2.6, SSP2-4.5, SSP5-8.5) as predicted by five GCMs. And the "Ens" is the ensemble mean of five GCMs. The graph indicates a consistent increase in average rainfall predicted by all five GCMs under each climate change scenario. The "Ens" for the predicted variations in the average annual rainfall over 20 years is highest at 13.1% in the mid-century and highest at 14.3% towards the end of the century. Notably, FGO predicts a substantial rainfall increase of 34.0% for the late-century under the SSP5-8.5 emission scenario. The five GCMs also predict an increase in the average annual temperature over multiple years. Among them, the "Ens" is highest at 1.8℃ in the mid-century and highest at 2.1℃ by the end of the century. Except for IPSL, which shows higher predicted increases under the SSP5-8.5 emission scenario (2.5℃ in the mid-century and 3.0℃ by the end of the century), the predicted increases from the other four models are relatively close.

**Figure 9:**
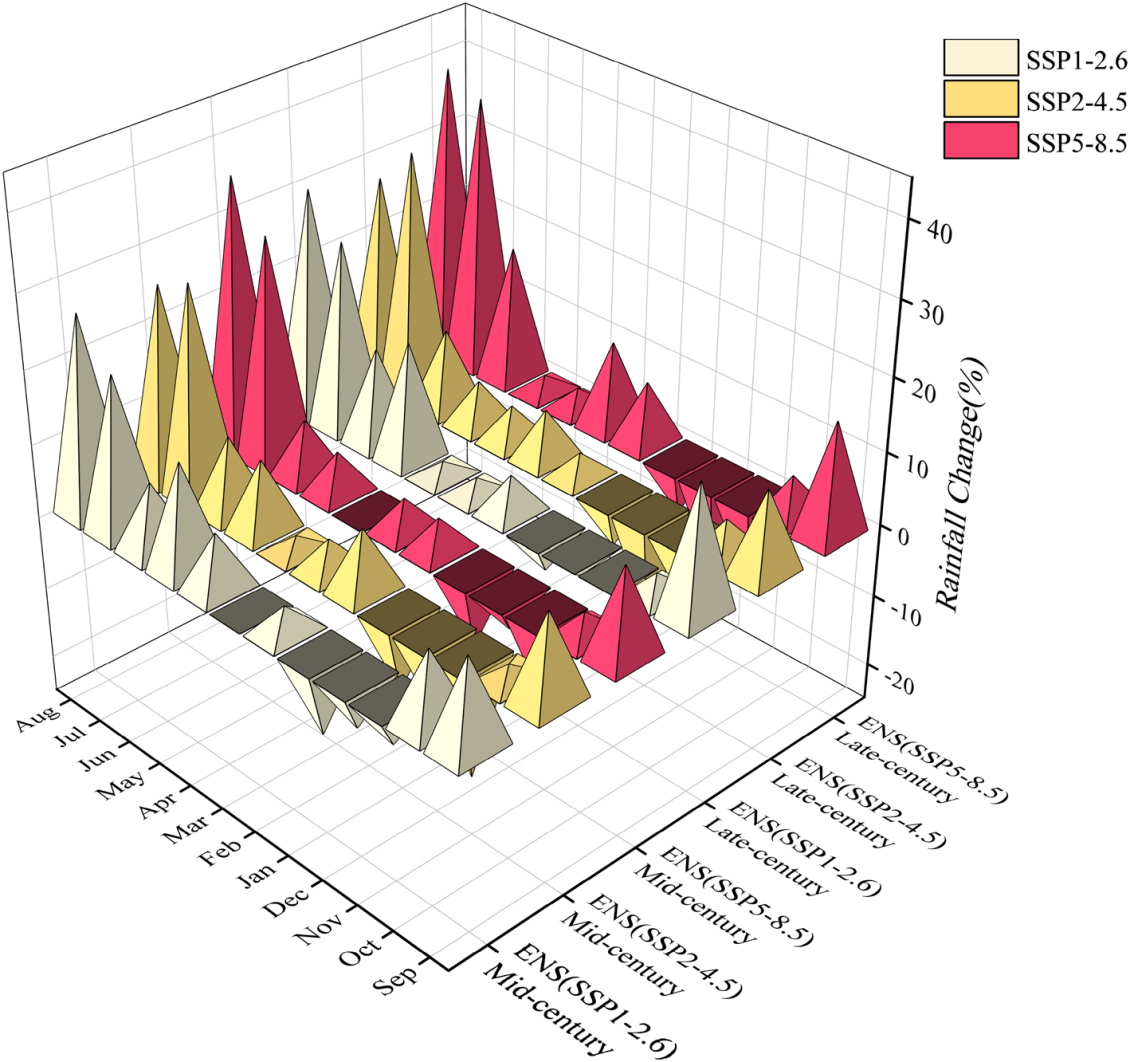
Relative change in monthly rainfall by the "Ens".

Figure 9 provides a comparative analysis of the "Ens" of monthly average precipitation changes projected by five Global Climate Models (GCMs) under various climate change scenarios. For both the mid-century and late-century periods, across all three emission scenarios, the projections indicate that out of the 12 months, seven months (April to October) will experience an increase in monthly precipitation, while only three months (November, December, and January) will see a decrease. It is noticeable that the highest increase in monthly rainfall occurs in July and August. In the mid-century, the rainfall in July increases by 20.8%, 28.2%, and 30.1% under three emission scenarios, while the rainfall in August increases by 26.5%, 25.8%, and 35.9%. By the end of the century, the rainfall in July increases by 25.2%, 33.1%, and 36.6%, while the rainfall in August increases by 30.2%, 27.8%, and 38.8% respectively. The HRB, situated in a temperate semi-humid and semi-arid continental monsoon climate zone, experiences prevailing north and northwest winds in winter, southeast winds in summer, and dry and windy conditions in spring. The region exhibits distinct wet and dry seasons, with rainfall contributing to 70–85% of the annual precipitation during the flood season (June to September). Major rainfall events are concentrated in July and August, making the region susceptible to flooding disasters. As the precipitation in these two months increases, the frequency of water-related hazards in the HRB will also rise, particularly in terms of floods. Furthermore, the windward slopes of the northwest arc mountains in the HRB, including the capital economic region encompassing Beijing, form a rainy belt with an annual precipitation exceeding 600 mm. With climate change, the risk of urban waterlogging in this area is expected to significantly increase.

#### Projected changes in streamflow

**Figure 10:**
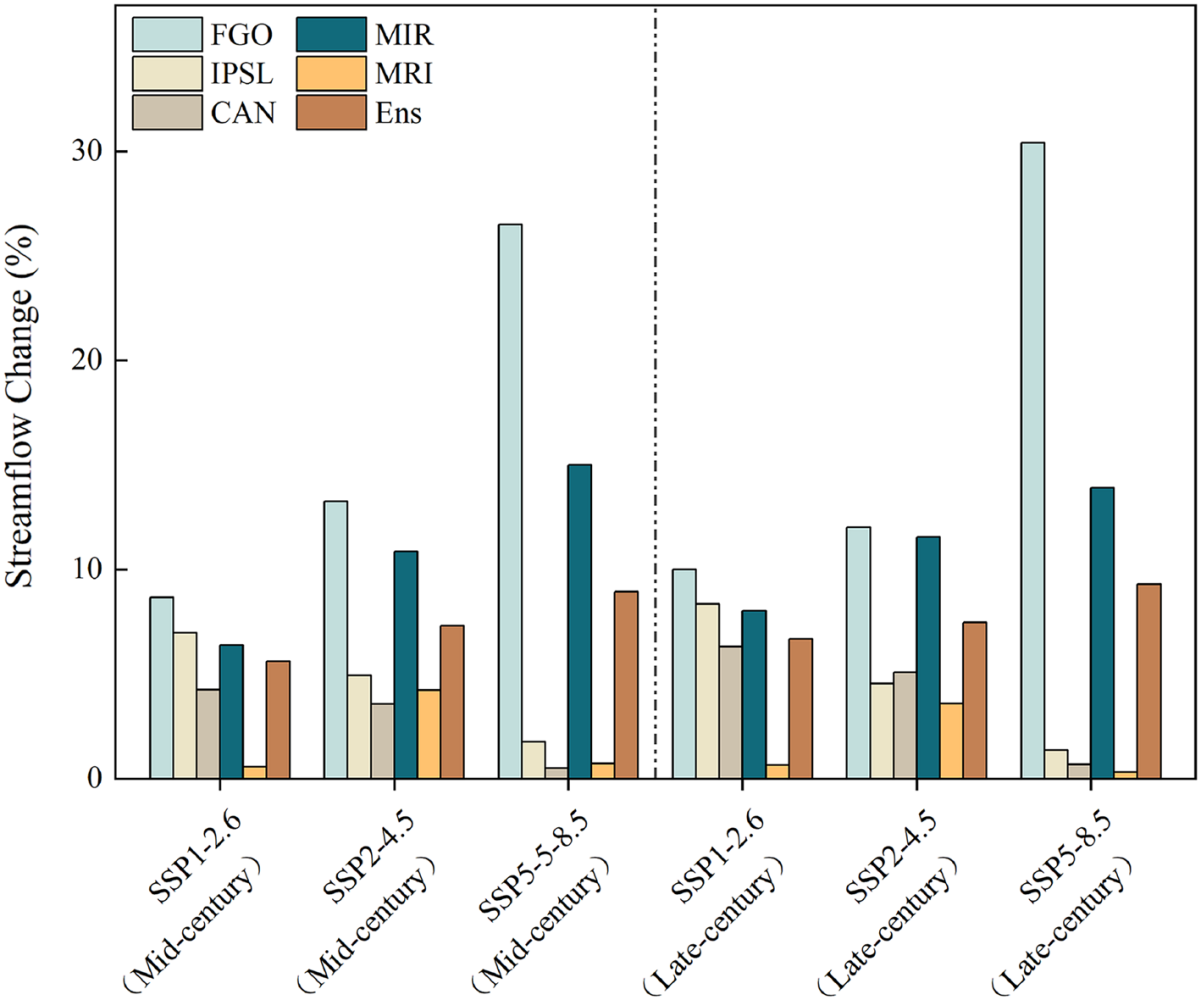
Relative change in multi-year average streamflow.

**Figure 11:**
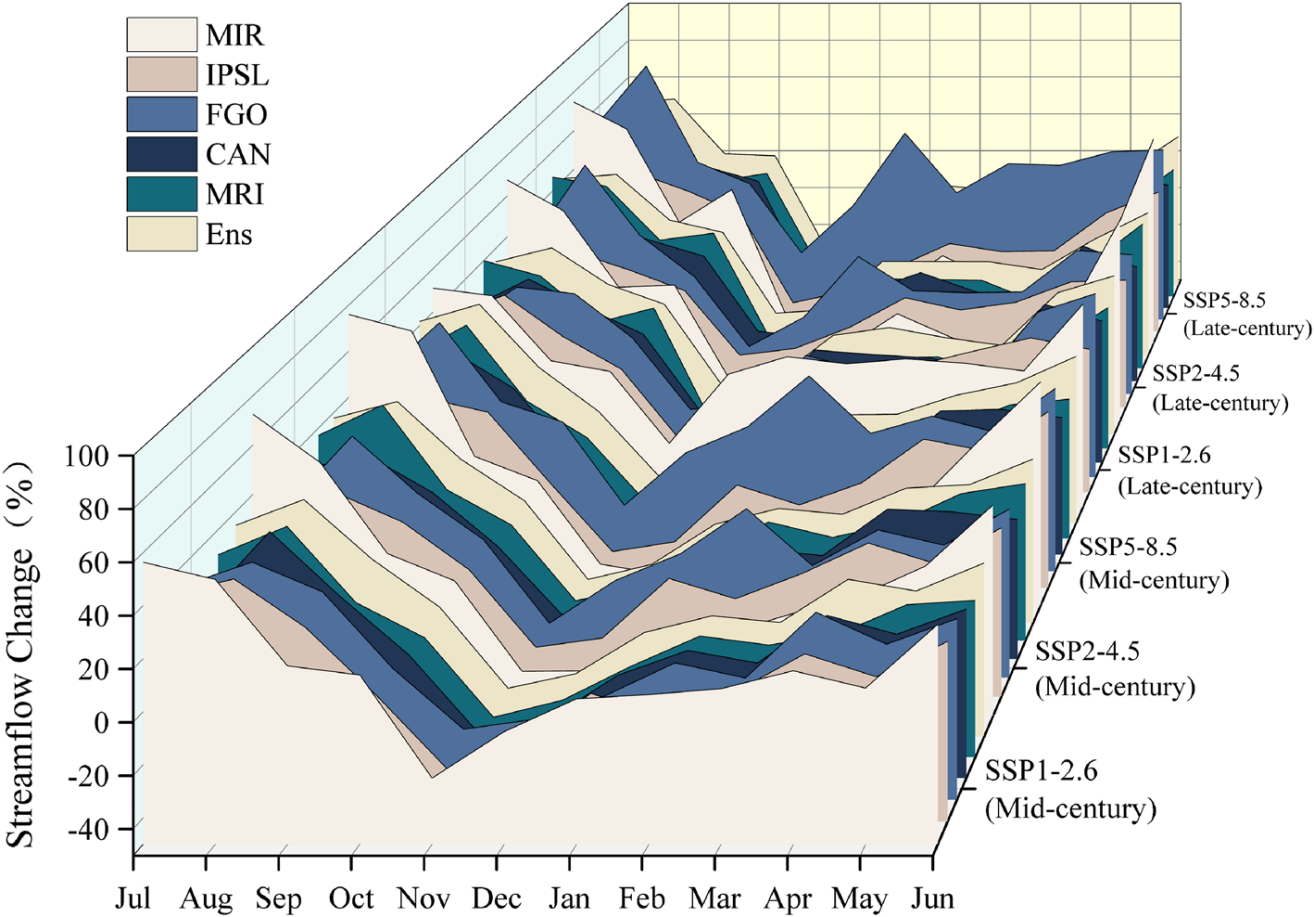
Relative change in monthly mean streamflow.

After downscaled processing using LarsWG on the monthly rainfall and temperature data generated by five GCMs under three emission scenarios for a total of forty years, the resulting daily meteorological forecast data is inputted into SWAT-C-BiLSTM with SinoLC-1 to simulate streamflow in the HRB. Figure 10 shows the predicted variations in the multi-year average streamflow at Zhangfang hydrological station (HRB outlet section) in the mid-century and end of the century relative to the baseline period (2002 to 2022). The analysis indicates an overall increase in annual streamflow across all climate change scenarios, albeit with varying magnitudes among different GCMs. The multi-year average streamflow predictions provided by FGO are the highest among the five GCMs under all three emission scenarios. In the SSP5-8.5 scenario, the predicted results for the mid-century and end of the century show an increase of up to 26.5% and 30.4% respectively compared to the baseline. In contrast, the predictions given by MRI are the lowest, with an increase of only 0.6% and 0.8% for the mid-century and end of the century respectively under the SSP1-2.6 scenario. For instance, in the late-century, the SSP5-8.5 scenario predicts an annual streamflow increase ranging from 0.3% to 30.4%, while SSP1-2.6 predicts an increase of 0.7% to 10.2%, and SSP2-4.5 predicts an increase of 3.6% to 12.0%. Overall, the "Ens" of predicted annual average streamflow changes ranges from 5.6% to 9.1% for the mid-century and from 6.7% to 9.3% for the late-century across the three emission scenarios. Figure 11 illustrates the predicted results of monthly average streamflow variations by five GCMs and their "Ens" under different emission scenarios. It can be observed that, in all three emission scenarios, the monthly average streamflow changes in the mid-century and end of the century exhibit similar patterns. They generally show an increase from April to October and a decrease from November to March. On average, across all three emission scenarios, it is projected that monthly streamflow will increase by 12% in May, 24% in June, 34% in July, 49% in August, and 21% in September. The increases in October and April are not expected to exceed 10%. However, from November to March, the results show a change in the streamflow direction, with relatively small absolute changes in streamflow volume, generally not exceeding 3%. Overall, the significant increase in monthly streamflow during the flood season (June to September), particularly in July and August, will significantly raise the frequency of flooding and urban waterlogging in the HRB, which deserves attention.

Although SWAT-C-BiLSTM with SinoLC-1 performs the best among all models, the changes in LULC over the next few decades are unpredictable. Modeling using VRH data can only provide the best simulated data of streamflow in the HRB under the current land surface environment as a reference.

## Conclusion

Conceptual hydrological models like SWAT face issues such as equifinality and uncertainty during parameter calibration, while data-driven models like BiLSTM may perform poorly when training data representativeness is low. By coupling SWAT and BiLSTM, it is possible to enhance the model's performance in simulating runoff and reduce calibration difficulty. Additionally, we used 1 m-resolution SinoLC-1 LULC data to optimize the delineation of HRUs and further improve the model performance. To validate the performance of the model, we set up the model in three different ways. First, we directly coupled the SWAT-D-BiLSTM model without calibrated parameters using default values. Second, we calibrated the parameters with P-values less than 0.4 within each sub-basin before coupling the SWAT-C-BiLSTM model. Finally, we used the SWAT-C-BiLSTM with SinoLC-1, which incorporated 1 m-resolution LULC data. The simulation results during the validation period (2018–2022) indicated that the performance of SWAT-C-BiLSTM with SinoLC-1 was superior to that of SWAT-C-BiLSTM, SWAT-D-BiLSTM, independent SWAT, and independent BiLSTM models. The Nash–Sutcliffe Efficiency (NSE) and R-squared (R^2^) values exceeded 0.92 during both calibration and validation periods. This suggests that partially calibrating the SWAT model and using VRH data can improve the applicability of the coupled model in large-scale watersheds with extensive geographical coverage and diverse watershed characteristics. It also helps to minimize the uncertainties associated with parameter calibration in the SWAT modeling process. This provides a promising framework for hydrological simulation in large-scale watersheds in other regions worldwide in the future. Particularly, with the development of computer technology, handling higher-precision data or using more advanced deep learning models will further enhance the performance of hydrological simulation in complex watershed features. The predictions generated by GCMs under three emission scenarios were utilized as input data to assess the influence of climate change on streamflow in the HRB. These predictions were incorporated into the analysis using the most effective model, SWAT-C-BiLSTM with SinoLC-1. The simulation results indicate that climate change will exacerbate the uneven spatial and temporal distribution of water in the HRB (Hydrological Response Basin) during the mid-century and end of the century. This will increase the risks of droughts, floods, and urban waterlogging. In the flood-prone months of July and August, with a significant increase in precipitation, the frequency and magnitude of floods will also rise. The existing flood control and drainage measures will be insufficient to cope with such magnitude of flood disasters, undoubtedly raising the risk of destructive floods in the HRB. It also reminds us to pay attention to appropriately raising the standards of flood control and drainage engineering measures during different stages of climate change to cope with future high-frequency flood disasters with large flow rates.

## Data Availability

The datasets used and/or analysed during the current study available from the corresponding author on reasonable request.
